# Clinical management and outcome differences between first and second waves among COVID-19 hospitalized patients: A regional prospective observational cohort

**DOI:** 10.1371/journal.pone.0258918

**Published:** 2021-10-28

**Authors:** María Zuil, Iván D. Benítez, Ramón Cabo-Gambín, Carlos Manzano Senra, Anna Moncusí-Moix, Clara Gort-Paniello, David de Gonzalo-Calvo, Marta Molinero, Jose Javier Vengoechea Aragoncillo, Thais Comella, Jordi de Batlle, Gerard Torres, Antoni Torres, Ferrán Barbé, Jessica González

**Affiliations:** 1 Group of Translational Research in Respiratory Medicine, IRBLleida, Hospital Universitari Arnau de Vilanova and Santa Maria, Centro de Investigación Biomédica en Red de Enfermedades Respiratorias (CIBERES), Lleida, Spain; 2 CIBER of Respiratory Diseases (CIBERES), Institute of Health Carlos III, Madrid, Spain; 3 Pulmonary Department, Hospital Clinic, Universitat de Barcelona, IDIBAPS, ICREA, Barcelona, Spain; Heidelberg University Hospital, GERMANY

## Abstract

The objective was to describe the clinical characteristics and outcomes of hospitalized COVID-19 patients during the two different epidemic periods. Prospective, observational, cohort study of hospitalized COVID-19. A total of 421 consecutive patients were included, 188 during the first period (March-May 2020) and 233 in the second wave (July-December 2020). Clinical, epidemiological, prognostic and therapeutic data were compared. Patients of the first outbreak were older and more comorbid, presented worse PaO2/FiO2 ratio and an increased creatinine and D-dimer levels at hospital admission. The hospital stay was shorter (14.5[8;29] vs 8[6;14] days, p<0.001), ICU admissions (31.9% vs 13.3%, p<0.001) and the number of patients who required mechanical ventilation (OR = 0.12 [0.05–10.26]; p<0.001) were reduced. There were no significant differences in hospital and 30-day after discharge mortality (adjusted HR = 1.56; p = 0.1056) or hospital readmissions. New treatments and clinical strategies appear to improve hospital length, ICU admissions and the requirement for mechanical ventilation. However, we did not observe differences in mortality or readmissions.

## Introduction

As noted in many countries worldwide, a two-wave pattern in reported cases of COVID-19 during the 2020 pandemic has been observed in Spain. Several randomized controlled trials have been conducted during the first wave, leading to antiviral treatment options and anti-inflammatory therapies that demonstrated better outcomes [1, 2]. Additionally, the experience gained during this period may have contributed to improving the management and outcomes in COVID-19 patients admitted during the second wave.

There is growing interest in evaluating the effect of these changes over months on mortality trends in clinical cohorts. Evidence in Europe tends to show that mortality from COVID-19 hospitalized patients was reduced in the second wave [3–6]. Nevertheless, inconsistent results are emerging [7], highlighting the need for well-characterized cohort analysis adjusting for confounding variables.

We therefore compared characteristics and outcomes between patients admitted to our hospital due to COVID-19 in Lleida (Spain) during the first wave (March to May 2020) and those admitted during the second wave (July to December 2020) to evaluate the effect of the different management practices on clinical outcomes and mortality in these patients.

## Methods

### Study setting and data collection

A prospective observational cohort study of hospitalized COVID-19 patients in Hospital Universitari Arnau de Vilanova and Hospital Universitari Santa Maria in Lleida (Spain) was performed. Both institutions follow the same protocols and work jointly. They are the reference center for a population of approximately of 450.000 people. The regular capacity is 600 beds and 38 ICU beds.

An emergence of a new variant of SARS-CoV-2 (20A.EU1) in early summer (end of June) in the north and east of Spain [8] was observed. This variant was linked to outbreaks among young agricultural workers in our region and transmission to the general population in that area was then replicated across the country and the rest of Europe. So, the period between March and May was considered as the first wave of the epidemic while from July to December, as the second wave of the outbreak of our region. This division is support by others studies around our territory [3, 9].

A total of 421 consecutive patients were included, of whom 188 were recruited during the first wave and 233 during the second wave. The regional strategy changed to adapt the epidemic peak based on clinical need and hospital situation. During the highest peaks of COVID-19, the hospital capacity expanded to the maximum possible, with 14 more ICU beds and reaching a maximum of 4 wards exclusively for COVID-19 in both periods.

All patients were aged over 18 years old and admitted to the general ward for respiratory infection due to SARS-CoV-2 virus. COVID diagnosis was confirmed by real-time reverse transcription polymerase chain reaction (RT-PCR) testing performed on nasopharyngeal throat swab specimens. There were no cases of nosocomial infection. Admission criteria to the hospital were to have a COVID-19 pneumoniae with one of the following severity criteria: respiratory rate > 20 breaths per minute or peripheral oxygen saturation <95% or PaO_2_/FiO_2_ ratio <300 or hemodynamic instability.

This study was approved by the local ethics committee (CEIC-2279). Informed consent was acquired (written and/or verbally) for all patients by using emergency consent mechanisms in accordance with the ethics approval guidelines for the study.

Patients’ sociodemographic characteristics, comorbidities, and clinical, vital, ventilatory and laboratory parameters were recorded at hospital admission. The length of hospital stay, administered treatments, respiratory support, bacterial coinfections, complications and in-hospital and 30-day mortality after hospital discharge or readmissions were recorded and compared between waves.

Pharmacological treatment of COVID-19 patients followed regional recommendations and protocols, based on the emergence of new evidence over time during the study period. Hydroxychloroquine was used from March to May 2020 while intermediate or full dose thromboprophylaxis was initiated on March 2020, metilprednisolone in bolus in May 2020 and remdesivir, tocilizumab and dexamethasone were initiated in June 2020.

### Statistical analysis

Cox regression models were used to predict mortality and ICU admission, and logistic models were used to predict the need for respiratory support. These models were adjusted based on age, sex and comorbidities.

## Results

Table 1 shows the comparison between the first and second waves regarding patient characteristics, comorbidities, biological data, clinical management and outcomes, such as complications, length of stay, ICU admissions, readmissions and mortality. Patients in the first wave were slightly older, had a higher prevalence of hypertension and chronic kidney disease and presented a worse PaO_2_/FiO_2_ ratio at admission. Additionally, these patients had higher levels of creatinine and D-dimer. No differences were observed in levels of C-reactive protein, lactate dehydrogenase or ferritin at admission.

**Table 1:** Clinical and outcome comparison between COVID-19 hospitalized patients admitted during the first (n = 188) and the second (n = 233) COVID-19 wave.

	First wave	Second wave	Difference		
	(n = 188)	(n = 233)			
	*Median (p*_*25*_ *;p*_*75*_ *) or n (%)*	*Median (p*_*25*_ *;p*_*75*_ *) or n (%)*	OR (95%CI)	p value	N
**Data at admission**					
*Patient’s characteristics*					
Age (years)	73.0 [61.0;84.0]	68.0 [57.0;80.0]	0.98 (0.97 to 0.99)	**0.006**	421
Male (sex)	72 (38.3%)	110 (47.2%)	1.44 (0.97 to 2.13)	0.068	421
Onset of symptoms to hospital admission (days)	7.00 [3.00;9.50]	7.00 [4.00;9.00]	1.01 (0.97 to 1.04)	0.784	392
*Main comorbidities*					
Obesity (BMI > 30 kg/m2)	60 (48.0%)	78 (39.0%)	0.69 (0.44 to 1.09)	0.113	325
Arterial hypertension	120 (64.5%)	126 (54.1%)	0.65 (0.44 to 0.96)	**0.032**	419
Diabetes mellitus	58 (31.0%)	54 (23.3%)	0.68 (0.44 to 1.04)	0.077	419
Ischemic cardiopathy	18 (9.78%)	13 (5.58%)	0.55 (0.25 to 1.15)	0.111	417
Chronic kidney disease	37 (20.0%)	28 (12.0%)	0.55 (0.32 to 0.93)	**0.027**	418
COPD / bronchiectasis	30 (16.0%)	26 (11.2%)	0.66 (0.37 to 1.16)	0.148	420
Immunocompromised status	7 (3.76%)	1 (0.43%)	0.12 (0.00 to 0.72)	0.017	419
*Analytical parameters*					
C-reactive protein (mg/dL)	107 [40.0;165]	88.5 [37.5;147]	1.00 (1.00 to 1.00)	0.071	397
Lymphocytes (x 10^9/L)	0.89 [0.63;1.25]	0.93 [0.68;1.39]	0.95 (0.87 to 1.04)	0.282	412
Creatinine (mg/dL)	0.92 [0.73;1.25]	0.87 [0.70;1.12]	0.67 (0.49 to 0.90)	0.009	410
D-dimer (ng/mL)	368 [230;864]	279 [208;472]	1.00 (1.00 to 1.00)	0.028	335
Prothrombin time (%)	1.17 [1.11;1.28]	1.15 [1.08;1.22]	0.87 (0.72 to 1.05)	0.148	396
Activated Partial Thromboplastin Time (seg)	30.1 [27.9;32.8]	29.8 [27.7;32.1]	0.97 (0.95 to 1.00)	0.056	396
Fibrinogen (g/L)	5.70 [4.90;7.00]	5.50 [4.80;6.20]	0.87 (0.76 to 1.00)	0.050	389
Platelets count (x 10^9/L)	202 [142;254]	184 [149;232]	1.00 (1.00 to 1.00)	0.323	407
Ferritin (mg/dL)	554 [270;1117]	593 [267;1048]	1.00 (1.00 to 1.00)	0.765	309
Lactate dehydrogenase (U/L)	616 [464;878]	611 [495;762]	1.00 (1.00 to 1.00)	0.149	294
*Arterial blood gas*					
pH	7.45 [7.42;7.47]	7.46 [7.43;7.49]	480 (8.80 to 26181)	0.002	355
PaO2 (mmHg)	62.0 [51.0;77.5]	65.0 [59.0;75.0]	1.00 (0.99 to 1.01)	0.787	356
PaCO2 (mmHg)	34.0 [31.0;39.0]	33.0 [31.0;38.0]	0.99 (0.97 to 1.02)	0.621	355
SatO2 (%)	94.0 [89.8;96.0]	95.0 [92.0;96.0]	1.04 (1.00 to 1.08)	0.056	357
PaO2/FiO2	252 [205;319]	290 [247;327]	1.00 (1.00 to 1.01)	0.004	348
**Clinical management**					
*Pharmacological treatment*					
Hydroxychloroquine	168 (89.4%)	1 (0.43%)	0.00 (0.00 to 0.00)	<0.001	421
Glucocorticoids	101 (54.3%)	202 (89.8%)	7.32 (4.42 to 12.6)	<0.001	411
Bolus administration	29 (16.4%)	5 (2.28%)	0.12 (0.04 to 0.30)	<0.001	396
Dexamethasone	0 (0.00%)	167 (71.7%)	···	···	421
Others	91 (48.9%)	34 (15.1%)	0.19 (0.12 to 0.30)	<0.001	411
Intermediate or full-dose thromboprophylaxis	160 (89.4%)	205 (93.2%)	1.62 (0.80 to 3.35)	0.184	421
Antibiotic therapy for bacterial co-infection	146 (78.1%)	95 (41.5%)	0.20 (0.13 to 0.31)	<0.001	416
Antiviral drugs	51 (28.2%)	61 (26.5%)	0.92 (0.59 to 1.43)	0.709	411
Lopinavir/Ritonavir	51 (27.7%)	1 (0.43%)	0.01 (0.00 to 0.06)	<0.001	416
Remdesivir	0 (0.00%)	61 (26.5%)	···	···	411
Tocilizumab	31 (16.5%)	120 (51.5%)	5.34 (3.39 to 8.60)	<0.001	421
*Procedures*					
High flow oxygen	42 (22.3%)	86 (36.9%)	2.03 (1.32 to 3.15)	0.001	421
Noninvasive mechanical ventilation	48 (25.5%)	42 (18.0%)	0.64 (0.40 to 1.03)	0.064	421
Orotracheal intubation	42 (22.3%)	10 (4.29%)	0.16 (0.07 to 0.31)	<0.001	421
Duration orotracheal intubation	24.0 [14.0;30.0]	26.5 [13.8;39.0]	1.02 (0.99 to 1.06)	0.196	47
Prone positioning	32 (17.0%)	13 (5.58%)	0.29 (0.14 to 0.56)	<0.001	421
**Outcomes**					
Hemofiltration	5 (2.70%)	0 (0.00%)	···	···	418
Deep vein thrombosis	4 (2.13%)	1 (0.43%)	0.22 (0.01 to 1.61)	0.146	419
Bleeding	5 (2.72%)	3 (1.29%)	0.48 (0.09 to 2.05)	0.320	417
Stroke	3 (1.60%)	3 (1.29%)	0.80 (0.14 to 4.71)	0.795	420
PE	1 (0.53%)	4 (1.72%)	2.97 (0.41 to 81.6)	0.309	420
Acute coronary syndrome	1 (0.53%)	0 (0.00%)	···	···	421
Arrhythmia	11 (5.95%)	6 (2.58%)	0.42 (0.14 to 1.15)	0.093	418
Shock	23 (13.1%)	7 (3.00%)	0.21 [0.08;0.48]	<0.001	408
Hospital stay (days)	14.5 [8.00;29.2]	8.00 [6.00;14.0]	0.96 (0.95 to 0.98)	<0.001	421
ICU admission	60 (31.9%)	31 (13.3%)	0.33 (0.20 to 0.53)	<0.001	421
Hospital stay (days)	36.0 [16.5;46.0]	24.0 [13.5;33.5]	0.99 [0.96;1.01]	0.156	91
In-hospital mortality	31 (16.5%)	31 (13.3%)	0.78 (0.45 to 1.34)	0.364	421
Hospital mortality	18 (14.1%)	23 (11.4%)	0.79 (0.39 to 1.62)	0.496	330
ICU mortality	13 (21.6%)	8 (25.8%)	1.25 [0.39;3.83]	0.793	91
30-days mortality	37 (19.8%)	37 (16.5%)	0.80 (0.48 to 1.33)	0.394	411
Re-entry (in the first 30 days)	16 (10.6%)	16 (8.29%)	0.76 (0.36 to 1.60)	0.471	344

BMI = body mass index; CRP: reactive protein; COPD = chronic obstructive pulmonary disease; PaO2: partial pressure of oxygen; PaCO2 = partial pressure of carbon dioxide; FiO2: Fraction of inspired oxygen; PE = pulmonary embolism; ICU = intensive care unit.

Contrary to the first wave, patients in the second period more frequently received tocilizumab, remdesivir, and dexamethasone as steroid therapy. During the second wave, the hospital stay was shorter, ICU admissions were reduced, and the number of patients who required invasive mechanical ventilation (adjusted OR = 0.12 [0.05–10.26]; p<0.001) and prone positioning were reduced (adjusted OR = 0.26 [0.12–0.56]; p = 0.001). High-flow oxygen therapy was more frequently used in the second wave (adjusted OR = 2.26 [1.34–3.91]; p = 0.003).

**Fig 1:**
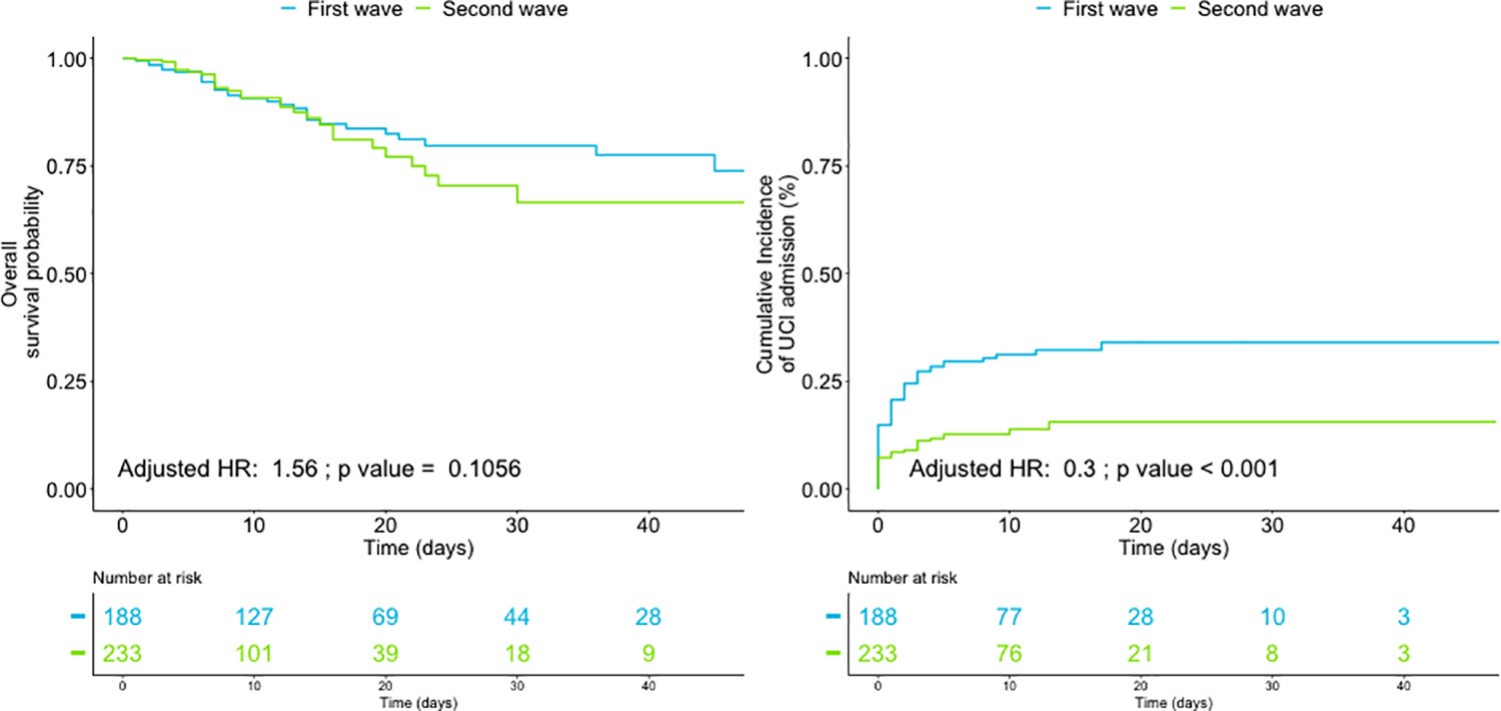
Survival analysis of overall mortality and ICU admission by waves. Adjusted HR = adjusted hazard ratio.

In-hospital complications, such as bacterial coinfection and septic shock, were also minimized. Adjusted analysis did not show significant differences in inpatient and 30-day after discharge mortality (Fig 1: adjusted HR = 1.56; p = 0.105) or hospital readmissions between waves. Mortality remain also unchanged between waves doing separated analysis focus in ICU (13 of 60 (21.6%) vs 8 of 31 (25.8%); p = 0.793) or in general ward patients (18 of 128 (14.1%) vs 23 of 202 (11.4%); p = 0.496).

### Associated data

Data is available on Figshare (10.6084/m9.figshare.16750480).

## Discussion

We report a study comparing the clinical characteristics and outcomes of hospitalized COVID-19 patients between the first and second waves in a well-characterized prospective cohort. Despite a shift towards younger and overall less comorbid individuals and apparent better management with substantial treatment modifications, we did not observe significant differences in ICU, inpatient and 30-days after discharge mortality or in number of readmissions.

These results contrast with others [3–5] but are consistent with a recent study performed in an ICU cohort in France [7]. The main difference is the increased mortality observed in the first wave in these studies [3–5, 10] ranging from 25 to 42%), which is drastically reduced in the second wave (range 7.3 to 26.9%) compared to ours (16.5% to 13.3%). Basically, these large studies are based on the administrative data of all patients admitted to hospitals with a lack of relevant variables for adjustment and with more susceptibility to hospital overload management. In fact, when stratifying by age, these differences disappear in some studies or remain only in patients aged above 70 years old (3). This finding could potentially be neutralized with a well-characterized and representative cohort and by performing adjusted analysis.

In general, all studies showed a reduction in hospital length stay with second wave patients being less likely to require ICU admission and mechanical ventilation. Improvements in medical skills, including the more frequent use of high-flow oxygen therapy within the last several months and the emergence of new treatments, such as antiviral and anti-inflammatory therapies [1, 2] are evident and could have an impact on these improved outcomes. However, despite the changes in evidence-based therapy over time, there is still a pool of patients that unavoidably end up developing a more severe disease with an acute distress respiratory syndrome and a fatal outcome [11]. This could contribute to keep mortality rates unchanged between periods and highlights the importance of deeply identify these patients and finding better specific treatment strategies for them.

The strengths of this study are the comprehensive and accurate data collection from a prospective cohort. The most important limitation is the single-center setting, which could affect the generalizability of the results. Moreover, and despite the well characterized and representative cohort, there could be a selection bias inherent in the context of a global COVID-19 pandemic that has implied adapting hospitals and clinical decisions to the needs of each moment. Additionally, we did not assess viral variants during the two different periods of the study. And finally, we did not assess the possible differences of the severity of ICU patients with clinical scores such as SOFA, SAPS II or APACHE.

In conclusion, hospitalized COVID-19 patient characteristics and their management differed between waves. New treatments and clinical strategies appear to improve hospital length, ICU admissions and the requirement for mechanical ventilation. However, no impact on real-life mortality trends was observed between these periods.
